# Knowledge and Perception of Rabies among School Children in Rabies Endemic Areas of South Bhutan

**DOI:** 10.3390/tropicalmed6010028

**Published:** 2021-03-02

**Authors:** Lungten Lungten, Sangay Rinchen, Tenzin Tenzin, Waraphon Phimpraphai, Michel de Garine-Wichatitsky

**Affiliations:** 1 Faculty of Veterinary Medicine, Kasetsart University, Bangkok 10900, Thailand; michel.de_garine-wichatitsky@cirad.fr; 2 National Polytechnique Institute of Toulouse, 31076 Toulouse, France; 3 City Veterinary Hospital and Satellite Laboratory, Dewathang, Samdrup Jongkhar 41001, Bhutan; 4 National Centre of Animal Health, Department of Livestock, Thimphu 11001, Bhutan; srinchen@moaf.gov.bt (S.R.); tenzinvp@gmail.com (T.T.); 5 ASTRE, University Montpellier, CIRAD, INRAE, 34000 Montpellier, France

**Keywords:** rabies, school children, knowledge, attitude, practice, education, Bhutan

## Abstract

Rabies is endemic in southern Bhutan and children are the frequent victims of dog bites. We surveyed the knowledge, attitude, and practices on rabies among school children in three schools located in southern Bhutan. A total of 701 students (57.9% female, 42.1% male) with an age range of 12–21 years (mean: 15 years) participated in the survey, of which 98.2% had heard about rabies. Most of the students demonstrated a good level of knowledge (59.7%) and a favorable perception towards rabies (57.7%). Multivariable logistic analysis revealed the relation between knowledge and the awareness campaign (OR:1.5, 95% CI: 1.1–2.1). Similarly, higher grades of students (OR:1.9, 95%CI: 1.3–2.9) and employed mothers of the students (OR: 1.6, 95% CI: 1.0–2.7) were associated with more favorable perceptions. However, some knowledge gaps were identified in this study, such as students not being able to clearly mention the susceptible hosts of rabies, transmission routes, clinical signs, and prevention and control options. Therefore, regular awareness programs on rabies are necessary among school children in Bhutan.

## 1. Introduction

Rabies is caused by infection with a lyssavirus and is one of the most important Neglected Tropical Diseases [1]. Rabies is transmitted mainly through dog bites and causes approximately 59,000 human deaths every year [2,3]. The disease is endemic in Asia and Africa and most of the victims are children (40%) under the age of 15 years. This zoonosis results in economic losses of up to 8.6 billion USD annually and about 3 billion people are at risk of infection [2].

Rabies is 100% fatal once clinical signs appear. However, timely use of vaccine and rabies immune globulin (RIG), and appropriate post-exposure prophylaxis (PEP), can effectively prevent a productive viral infection [3]. Unfortunately, PEP is not easily accessible, especially to poor people and to remote rural communities in rabies endemic countries. Even when biologics are available, bite victims may not have the means to pay for transport to the hospital and cover the costs for PEP [2]. Inefficient health-seeking behavior of dog bite victims, such as seeking the assistance of traditional healers for local treatment at home, is also associated with a low level of knowledge and awareness about the health risk of rabies [4,5].

In Bhutan, rabies is endemic in the southern part of the country that shares a border with India, and reports around 17 outbreaks every year [6]. Sporadic outbreaks are also reported from the eastern parts of the country [7,8]. Dog bites are common. Approximately 7000 bite incidents are reported every year in the country, with a population of 700,000 people. Annually, the government spends approximately Nu 9.3 million (USD 142,000) on PEP- [9,10]. Seventeen human rabies death were reported between 2006 and 2016 [11]. One death was reported during 2020 in a three-year old child [12].

In Bhutan, PEP is provided free of charge to dog bite victims through a network of 240 health centers located across the country [9,11]. The general level of knowledge and awareness on rabies is thought to be high among the communities, which has been attributed to regular awareness programs [7,9,10,13]. However, children under the age of 15 years are at higher risk of experiencing dog bites and rabies deaths in Bhutan [10,14]. Little is known about their perceptions on rabies. As such, is important to understand the knowledge, attitudes, and practices (KAP) of school children regarding rabies and dog bites to design efficient prevention programs. In this study, we described the findings of a KAP survey on rabies among children in three secondary schools located in the rabies endemic zone of southern Bhutan and discuss possible prevention measures.

## 2. Materials and Methods

### 2.1. Study Area 

**Figure 1:**
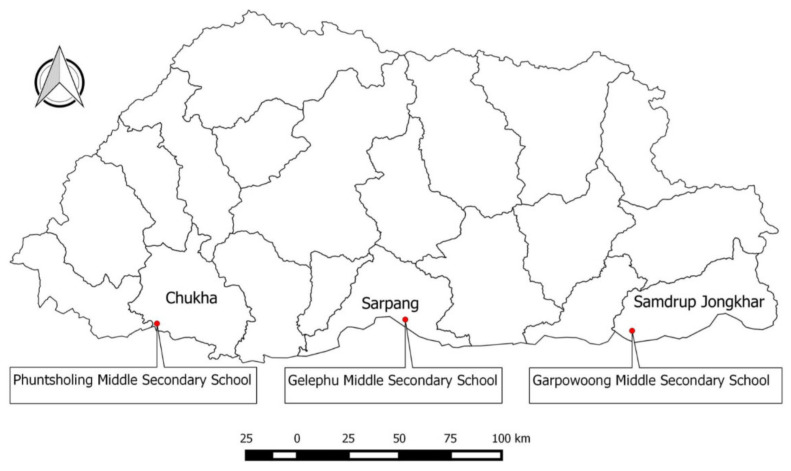
Map of Bhutan showing the location of three schools in which the study was conducted (Bhutan is located between China in the north and India in the south, east, and west). The names and borders of the districts are indicated as well as the location of the schools surveyed (red dots). The map was prepared using Quantum GIS, QGIS Development Team (2019), QGIS Geographic Information System, Open-Source Geospatial Foundation Project (http://qgis.osgeo.org) and was not taken from another source.

The study was conducted in the three towns of southern and eastern Bhutan sharing a border with India: Phuntsholing, Gelephu, and Samdrup Jongkhar (Figure 1). These areas report frequent outbreaks of animal rabies [6] and have recorded a greater incidence of dog bites compared to the rest of the country [9,14]

Approximately 44,328 people live in the three towns [15]: Phuntsholing (27,658), Gelephu (9858), and Samdrup Jongkhar (10,545). There are 14 schools and approximately 7809 students studying in these towns [15]. Of these, six are Higher Secondary Schools, four Middle Secondary Schools (MSS), two Lower Secondary Schools, and two Primary schools. Four Higher Secondary Schools from these three areas are private schools. The rest are government public schools.

### 2.2. Study Design and Data Collection 

In Phuntsholing, Phuntsholing Middle Secondary School (PMSS) was selected based on access and convenience, because the school is located in the core town area and has a high-risk of dog bites due to the large number of free-roaming dogs in the town. In Gelephu, since there is only one MSS, Gelephu Middle Secondary School (GMSS) was included in the study. Similarly, from Samdrup Jongkhar, of the two MSS, Garpowoong Middle Secondary School (GaMSS) was selected at random for the study. Assuming that grade 8, 9, and 10 students understand and correctly interpret the questionnaires, they were invited to participate in the study. Data were collected using an individual structured questionnaire. The questionnaire consisted of three sections:

1. demographic details of students;
2. knowledge, attitude, and practices of students regarding rabies;
3. dog bite incidence and health-seeking behavior of the respondent students.

Before the actual survey, the questionnaires were pre-tested with 10 students of GaMSS and changes were made to improve the clarity of the questions. All students in grades 8, 9, and 10 from the selected schools were enrolled in the study. Prior to school visits, approvals were obtained from both the school principals and class teachers. Before the actual data collection, students were explained the purpose of the study, that participation was voluntary, and they were allowed to withdraw at any stage. After obtaining verbal and written consent from the students, questionnaires were distributed for self-administration. The questionnaires were explained to the students and they were guided in answering each question.

### 2.3. Data Management and Analysis 

The questionnaire survey data were entered into a database developed in EpiInfo software version 7.2.3.1 [16]. The data were then extracted into Microsoft excel 2013 (Microsoft Excel, Redmon, WA, USA) and checked for any errors before performing the analysis (Table S1). Data analysis was performed with R statistical software version 3.6.1 using packages “dplyr”, “descr”, “forcats”, “LogisticDx”, and “ggplot2” [17]. Descriptive statistics were obtained by calculating the proportions, frequency, mean, median, standard deviation, and ranges. For analysis, variable age was categorized as “adolescent” for those students whose age was more than 15 years and “young” for those whose age was less than or equal to 15 using the mean age. The number of dogs owned by the students was categorized as “1 dog” if they owned only one dog at home and “more than 1 dog” if the students reported that they owned more than one dog at home. The occupation of the parents of the students was collapsed into two categories using the “forcats” R package. Those working in the military, government offices, and corporations were categorized together as “employed” and those that were working in business, as farmers, and others were considered as “self-employed.” Frequencies of the categorical variables related to socio-demographic characteristics, dog ownership status, and dog bite incidence were compared between the three schools using Pearson’s Chi-squared test with Yates’ continuity correction and Fisher’s Exact Test.

The knowledge of the students was assessed on sources of rabies, mode of transmissions, signs shown by rabid animals, and the preventive measures of rabies. For every correct answer (i.e., in agreement with the conventional medical knowledge on rabies), a score of “1” was allotted, and “0” was allotted for the wrong answers. The scoring method used is described in the Supplementary Materials (Table S1). Knowledge was based on the scores that students obtained in identifying rabies virus sources (1 point), susceptible hosts (5 points), mode of transmission (3 points), clinical signs (4 points), and rabies prevention measures (3 points). A maximum of 16 points was obtained if the students correctly answered all questions. The total scores obtained by each student were calculated and the total scores were categorized into binary outcome variables using the mean score of knowledge [18,19]. The students that had a knowledge score higher than the mean (≥7) were considered as “Good” and those that had a lesser knowledge score than the mean (<7) were considered as having “Poor” knowledge on rabies. Similarly, for the perception related questions, a score of “1” was allotted for the correct answers and “0” for the wrong answers. Perceptions were assessed on what students would do if they were bitten by dogs (3 points) and what they would do if they saw a dog with abnormal behavior (2 points). A maximum of five points were obtained if students correctly answered all the questions. If the perception scores were more than or equal to the mean (≥3.5), they were considered as “Favorable,” and if scores were less than the mean score (<3.5), they were considered as an “Unfavorable” perception to rabies.

Logistic regression models were built separately for student knowledge and perception (binary outcome variables) to analyze if there was any association with the explanatory variables: socio-demographic characteristics, dog ownership status, dog bite incidence, and education status of the students (whether they have attended rabies awareness program previously). The explanatory variables that had a *p*-value ≤ 0.20 in the univariable analysis were selected and used for the multivariable analysis. Only those variables that had *p* value ≤ 0.05 were retained in the final model. The goodness-of-fit for the model was assessed using the Hosmer Lemeshow test.

### 2.4. Ethical Approvals 

The study was approved by the Research Ethics Board of Health, Ministry of Health (Ref. No. REBH/Approval/2019/113). Administrative approvals were also obtained from the city education officers, and from the three school principals and class teachers prior to the study.

## 3. Results

### 3.1. Socio-Demographic Characteristics of the Students 

Of the 712 students invited to participate in the study, 701 students completed the survey (98.0%). Data from these completed questionnaires were used for analyses. The final group of students who completed the questionnaires was comprised of 406 (57.9%) females and 295 (42.1%) males. The students’ age ranged from 12 to 21 years (median: 15 years). The participants included 234 (43.4%) students from PMSS, 237 (33.8%) from GaMSS and 160 (22.8%) from GMSS. Most study participants were studying in grade 9 (*n* = 291, 41.5%), followed by grade 10 (*n* = 256, 36.5%) and 8 (*n* = 154, 22.0%). Most students (*n* = 490, 69.90%) resided in town with their parents and attended school as day-scholars. The socio-demographic details of the students are described in Table 1.

Among the 701 participants, 31.0% of the students owned dogs at their house (*n* = 217), with each household owing an average of 1.6 dogs. The proportion of households with dogs was significantly different between the schools (χ^2^ = 17.5, df = 2, *p*-value < 0.001), highest for students from GMSS (*n* = 83, 38.0%) and lowest in PMSS (*n* = 63, 29.0%). The students reported that the dogs that they owned were mostly given to them by neighbors (*n* = 115, 53.0%), were vaccinated (*n* = 163, 75.1%), and were allowed to roam freely (*n* = 121, 55.8%). Vaccination status of the owned dogs was significantly different among different schools (*p* = 0.009) with more vaccinated dogs reported by the dog owning students from PMSS (*n* = 67, 30.9%). Only one-third (*n* = 74, 33.6%) of the students reported that their dogs were sterilized/neutered (Table 2).

### 3.2. Dog Bites Incidence and Health-Seeking Behaviour 

The study found that 111 students (15.8%) had experienced dog bites in the last two years prior to the study period. Most bites were sustained from pet dogs (*n* = 58, 52.3%) compared to stray dogs (*n* = 44, 39.6%) with five students (*n* = 5, 4.5%) unable to ascertain the status of dog that bit them. Thirty students (50.0%) reported bites by pet dogs belonging to their neighbors. Most bite victims (*n* = 64, 57.7%) reported that they were bitten without any disturbance or provocation of the dog. Most unprovoked bites were reported from PMSS (*n* = 35, 31.5%), which is significantly higher than other two schools (*p* = 0.006). Regarding the care given after bites, the majority of students indicated that they washed the bite wound with soap and water (*n* = 69, 62.2%), visited hospitals (*n* = 49, 84.7%) and received PEP (*n* = 92, 82.9%). However, adoption of risky practices were also reported, such as application of local medicine, not visiting hospitals (*n* = 13, 11.7%), and non-completion of the complete vaccine schedule (*n* = 3, 3.2%). When asked about the fate of the dog that had bitten them, the majority of the victims (*n* = 56, 50.5%) reported that the dog was still alive, while twenty-three students (20.7%) could not ascertain the status of the dog and eighteen students (16.2%) reported that dog had died, but were unable to say if the dog had died because of rabies or other diseases (Table 3).

### 3.3. Students’ Knowledge Regarding Rabies 

**Figure 2:**
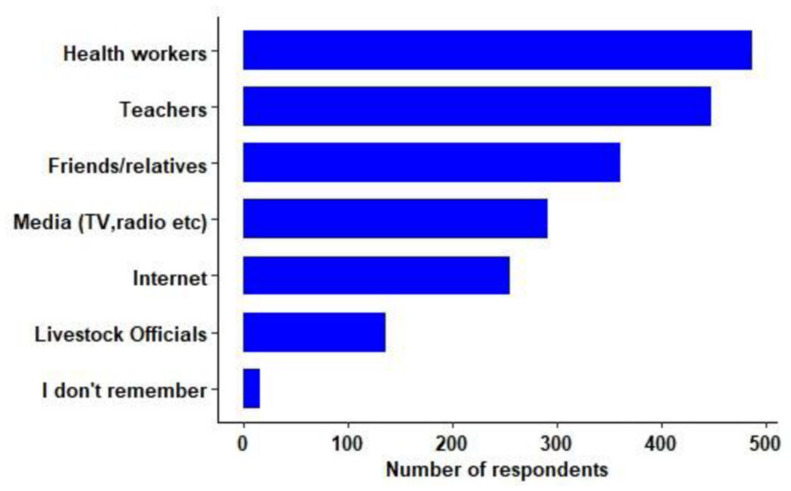
Sources of rabies information for the students of three secondary schools located in rabies endemic areas of south Bhutan (Phuntsholing, Gelephu, Garpowoong; *n* = 701).

Most students had heard about rabies (*n* = 688, 98.2%). The sources of information (Figure 2) were from health workers (*n* = 488, 70.9%), teachers (*n* = 449, 65.3%) and friends (*n* = 362, 52.6%). Among those who had heard about rabies, the majority (*n* = 622, 90.4%) knew that dog is the main source of rabies in Bhutan. However, few students mentioned that other animals, such as bats (*n* = 36, 5.2%), cats (*n* = 3, 0.4%), cows (*n* = 1, 0.1%) and birds (*n* = 2, 0.3%), are the main source of rabies. 

**Figure 3:**
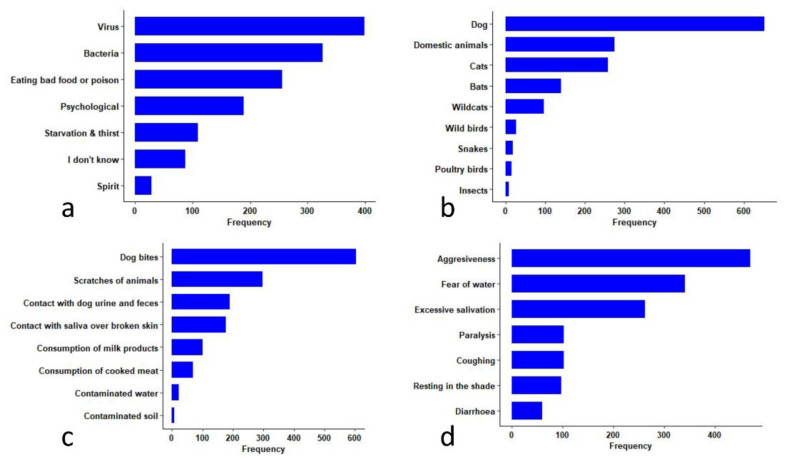
Knowledge on rabies among the students from study areas in South Bhutan: (**a**) knowledge on causes of rabies mentioned by the students; (**b**) knowledge on the susceptible hosts of rabies mentioned by the students; (**c**), knowledge on the mode of transmission of rabies mentioned by the students; and (**d**) knowledge on the clinical signs of rabies mentioned by the students. Each bar in the graph represents frequency of the positive responses by total students who heard about rabies (*n* = 688) to the specific cause, hosts, routes, and clinical signs of rabies.

Regarding the causes of rabies (Figure 3a), most of the students (*n* = 399, 58.0%) correctly identified viruses as the cause of rabies (Table 4), although some students associated rabies to other factors such as bacteria (*n* = 326, 47.4%), eating food or poison (*n* = 256, 37.2%), psychological problems (*n* = 189, 27.5%), starvation and thirst (*n* = 109, 15.8%), and Spirits (*n* = 28, 4.1%).

When asked about the susceptible host of rabies, almost all students (*n* = 651, 94.6%) mentioned that dogs are the main susceptible host for rabies. They also correctly identified other domestic mammals like cattle, pigs, and horses (*n* = 275, 40.0%), domestic cats (*n* = 256, 37.5%), bats (*n* = 140, 20.4%), and wild cats, such as tigers and leopards (*n* = 97, 14.1%) as susceptible hosts for rabies. However, some of the students surveyed answered that wild birds (*n* = 27, 3.9%), snakes (*n* = 19, 2.8%), poultry (*n* = 16, 2.3%), and insects (*n* = 9, 1.3%) can also be affected by rabies (Table 4, Figure 3b), which is not in agreement with conventional veterinary knowledge. When asked if rabies can infect humans, 636 (92.4%) students knew that humans could be infected, but 397 (57.7%) students were not able to ascertain the fatal nature of the disease when symptoms appear.

Regarding the transmission of rabies virus, 603 (87.7%) students understood that rabies can be transmitted through dog bites. Other possible transmission routes such as scratches due to animals and contact with saliva over broken skin were mentioned by 297 (43.2%) and 176 (25.6%) students, respectively. Transmission routes through consumption of milk products (*n* = 101, 14.7%), cooked meat (*n* = 68, 9.9%), contact with dog urine and feces (*n* = 190, 27.6%), contaminated water (*n* = 23, 3.3%) and contaminated soil (*n* = 8, 1.16%) were also reported (Table 4, Figure 3c), although these transmission routes are not in agreement with conventional veterinary knowledge.

Regarding clinical signs of rabies (Table 4, Figure 3d), most of the students mentioned that a rabid animal is aggressive (*n* = 470, 68.3%), fears water (*n* = 341, 49.6%), and excessively salivates (*n* = 262, 38.1%). Signs that were not usually associated with rabies, such as coughing (*n* = 102, 14.8%) and diarrhea (*n* = 60, 8.7%) were also reported by some students.

Most students knew that rabies in dogs can be prevented by vaccination (*n* = 452, 65.7%). Regarding the frequency of vaccination, 560 (81.4%) students mentioned that dogs should be vaccinated every year, while 105 (16.3%) students did not know the required frequency of vaccination. Few students (*n* = 20, 2.9%) mentioned that only one-time vaccination was needed in the dog’s lifetime, while three students (0.4%) mentioned that vaccination was not necessary. Other methods, such as preventing dogs from contacting stray dogs (*n* = 67, 9.7%), washing the dog with shampoo (*n* = 67, 9.7%), not allowing the dogs to feed on garbage (*n* = 54, 7.9%), and regular deworming (*n* = 19, 2.8%), were also mentioned by some students as preventive measures for rabies.

### 3.4. Students’ Perception towards Post-Bite Care and Rabid Dogs

**Figure 4:**
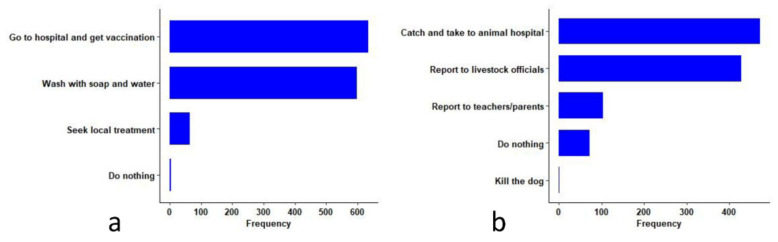
Students’ perceptions on rabies: (**a**) what they would do if they were bitten by a rabid dog; (**b**) what they would do if they saw rabid dog in the streets. Each bar in the graph represents frequency of the positive responses by total students who heard about rabies (*n* = 688) to the specific cause, hosts, routes, and clinical signs of rabies.

The details of what students would do if they were bitten by rabid dogs and what they would do if they saw a rabid dog in the streets are illustrated in Table 4 and Figure 4. Most students reported that they would wash the bite wound with soap and water (*n* = 598, 86.9%) and go to the hospital to get PEP (*n* = 635, 92.3%). However, risky practices, such as applying local medicines only (*n* = 65, 9.5%) and doing nothing (*n* = 5, 0.7%) to the bite wound were also reported. If they saw a dog suspected of rabies in streets, 472 (68.6%) students indicated that they would try to catch the dog and take it to the animal hospital for treatment. Attitudes for other behaviors, such as reporting to the livestock officers (*n* = 427, 62.1%), reporting to their teachers (*n* = 104, 12.0%), doing nothing (*n* = 72, 10.5%), and killing the dogs (*n* = 23, 3.3%) were also reported.

### 3.5. Logistic Regression Analyses

**Figure 5:**
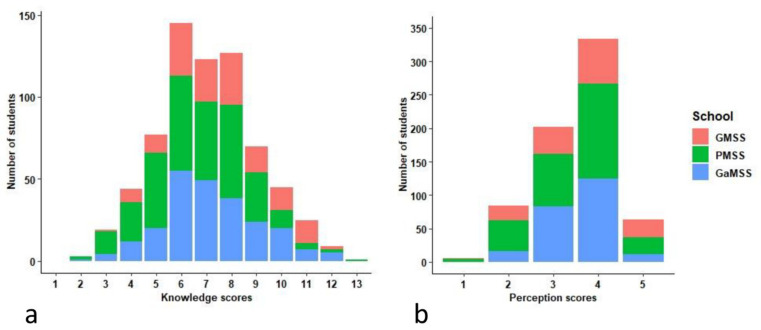
Distribution of knowledge and perception scores among students of three selected school: (**a**) Knowledge scores; (**b**) Perception scores.

The results of the univariable and multivariable analysis of factors associated with the knowledge and perception of the students are presented in Table 5. The mean knowledge score of the students was 7.1 (SD = 2.0). The distribution of knowledge scores among the students from the three schools are given in Figure 5a. Significant differences were observed in the knowledge scores of the students between the three different schools, with students of GMSS (mean score = 7.6) scoring higher than students of GaMSS (mean score= 7.2) and PMSS (mean score = 6.6) (*p* < 0.001). Using the cut-off score of ≥7 (mean), 411 of 688 (59.7%) students were classified as having a “good” knowledge score and 277 (40.3%) as having a “poor” knowledge score on rabies. The final model, after adjusting for sex, indicated that students who had attended rabies awareness programs prior to the survey (Adjusted Odd Ratio: 1.5, 95% CI: 1.1–2.1) had higher odds of having good knowledge compared to those that had not attended such a program. Similarly, students who were studying in grade 9 (AOR:1.1,95% CI: 0.8–1.8) and grade 10 (AOR:1.7. 95%CI:1.1–2.6) were more likely to have a good rabies knowledge compared with students studying in grade 8. Among the schools, students from PMSS (AOR: 0.7, 95% CI: 0.4–1.5) and from GaMSS (AOR: 0.5, 95% CI: 0.3–0.8) were less likely to have a good level of rabies knowledge compared to the students of GMSS.

The mean perception score was 3.5 (SD: 0.8, median: 4) with a minimum score of 1 and a maximum of 5. No significant differences were observed in the perception scores by the students among the three schools (*p* = 0.327). The mean scores of each class in different schools are presented in Figure 5b. Using the cut-off score of ≥3.5 (mean) to classify into a “favorable” and “unfavorable” perception score, 397 of 688 (57.7 %) students were classified as having a favorable perception on rabies, and 291 (42.3%) as unfavorable. After adjusting for sex and school, favorable attitudes and perceptions towards rabies were found significantly associated with grades (higher for grade 10; AOR: 1.9, 95%CI: 1.3–3.2) and the mother’s occupation (higher for employed mother; AOR: 1.7, 95%CI: 1.0–2.8). 

## 4. Discussion

Our study indicated that most students (98%) surveyed in rabies endemic areas of the country have heard about rabies and have a good level of knowledge, including the source of the disease, susceptible hosts, route of transmission, clinical signs, and preventive measures. This high awareness among students may be due to frequent reports of rabies outbreaks in the study areas, in addition to the rabies awareness campaigns conducted by the government. In Bhutan, the animal and public health officials conduct an annual education campaign related to dog bite prevention and rabies, coinciding with World Rabies Day (28th September). In addition to the general awareness program broadcasts by radio and television on rabies, the specific awareness education program is also organized in the schools. The school children also take part in the street walk in rabies endemic areas disseminating messages on the importance of dog vaccination and prevention of dog bites, among others. With most of the students citing health officials and teachers as important sources of knowledge about rabies, our findings indicate the benefits of rabies awareness programs in schools. However, the role of the teachers as information disseminating agents particularly for school children is not well recognized. In the current situation, they are rarely engaged in rabies preventive and control measures other than providing the logistic support during rabies awareness programs in their schools. Therefore, inclusion of teachers as one of key stakeholders in future rabies preventive and control measures would help to improve the level of knowledge of school children on rabies. The knowledge on rabies among students in this study is comparable to the adult population in the country [7,10,13] but higher than students from the neighboring Sikkim state of India (81%) studying in similar grades [20]. Although a majority of the students had a good level of general knowledge on rabies, some important gaps were identified (Table 2). The students usually did not know the fatal nature of the disease, that dogs are the most important sources of rabies, and that they must visit a hospital for PEP following dog bites, as local treatments are not efficient to prevent the disease. Studies conducted among students in India [20,21], Sri Lanka [22]), the Philippines [23], and Nigeria [24] have reported similar knowledge gaps, underlining the associated risks and the need for the specific rabies education in school children in rabies endemic areas. The knowledge and awareness of rabies was reported to be associated positively with several factors including the age of the respondents [25], sex [26,27], education level [20], dog ownership status [28], economic status [10], and religion [29]. Our study results showed that rabies knowledge in endemic areas of Bhutan was associated with the education level (grades) of the students. This result was expected because students studying in higher grades tend to have greater academic knowledge and a better understanding of the subjects in biology and health compared to lower grades. Similar observations were also reported for school children in the Sikkim State of India [20]. Among the three selected schools in southern Bhutan, students of GMSS had a better knowledge than the two other towns, which may be associated with high incidence of rabies in animals, dog bites, and PEP events in Gelephu [10,14], and a higher percentage of dog ownership [30]. A KAP study conducted among the adult population in Gelephu also demonstrated a higher understanding and knowledge of rabies compared to other areas of the country [10]. The higher level of knowledge demonstrated by students who had attended the rabies awareness program also indicated the importance of these programs as a means of rabies information dissemination, particularly for school children. As discussed previously, rabies awareness programs are provided to students on World Rabies Day, and also following rabies outbreaks. Improvement in knowledge after rabies education has been reported in other countries [20,22,23,28,31].

Globally, dog bites are responsible for more than 99% of rabies cases [3]. Therefore, thorough washing of bite wounds with soap and water and appropriate administration of PEP (including infiltration of RIG) are the only efficient methods to prevent rabies after a bite by a rabid dog [3,32]. Our study showed that most of the students had a favorable attitude and perceptions towards post-bite care and management. However, 13 students (11.7%) who had been bitten by dogs before our study had not visited the hospital and only sought local treatment (Table 3). This is of great concern since it would prove fatal if bitten by a rabid dog. Most of the human mortalities in rabies endemic countries occur following inefficient health-seeking behaviors by dog bite victims, who cannot, or do not want to, access appropriate medical treatment and resort to local treatments [4,19,21,33,34]. Furthermore, the students in our survey mentioned that they would try to catch and take a sick dog to a veterinary hospital for treatment. Although it is a good attitude on animal welfare grounds, this will put children at risk of contracting rabies. Therefore, it is important to educate children on the health risks of such practices and make them aware of whom to report the incidents in their locality or school premises. Our study also showed that children whose mothers are formally employed displayed a more favorable attitude towards post-bite care and rabid dogs. Possible reasons could be due to easy access to the rabies education materials by employed mothers, who might share with their children, since they may have greater education qualifications compared to some self-employed mothers. However, differences in roles played by employed mothers and self-employed mothers and their nature of interaction with the children need to be explored further, which can be used strategically in planning future rabies education programs. 

In addition to the KAP of students on rabies, we also collected data/information related to dog ownership in the study areas. Among various methods to estimate dog population, interviewing school children is also one of the methods to estimate the dog population and vaccination coverage [35]. Our survey found that 31.0 % (*n* = 217) of the students owned one or more dogs at home, which agrees with previous community studies conducted in Bhutan [30]. Although most of the students reported that the dogs they owned were vaccinated (73.0%), dog management appeared poor since most dogs were allowed to roam freely. Mixing with unowned and free-roaming dogs increases the risk of rabies virus transmission and can result in an increase in dog populations through uncontrolled reproduction.

## 5. Conclusions

Overall, our study in rabies endemic areas of Southern Bhutan showed that most students have a good knowledge and a favorable perception towards rabies. However, some knowledge gaps and unfavorable perceptions were identified that could put school children at risk of rabies acquisition. Therefore, regular rabies awareness programs is needed, particularly for school children who are studying in lower grades. In a limited resource setting, priority should be given to schools that are in an area where there is a greater number of rabies cases and a low level of knowledge (e.g., Phuntsholing and Samdrup Jongkhar). Moreover, our study also highlights the important role that could be played by school teachers in dissemination of rabies information for inclusion in future rabies preventive and control programs.

## Figures and Tables

**Table 1:** Socio-demographic characteristics of the students that participated in the study from three middle secondary schools located in rabies endemic areas of Bhutan (PMSS: Phuntsholing Middle Secondary School; GMSS: Gelephu Middle Secondary School; GaMSS: Garpowoong Middle Secondary School).

		School Name (*n* (%))			
Variables	Total (*n* %)	GMSS	PMSS	GaMSS	χ^2^ *p*-Value
Sex					0.258
Male	295 (42.1)	59 (8.4)	129 (18.4)	107 (15.3)	
Female	406 (57.9)	101 (14.4)	175 (25.0)	130 (18.5)	
Age					0.015
Young (≤15 yrs)	470 (67.0)	111 (15.8)	217 (31.0)	142 (13.6)	
Adolescent (>15 yrs.)	231 (33.0)	49 (7.0)	87 (12.4)	95 (13.6)	
Grade in which student study					<0.001
Grade 8	154 (22.0)	42 (6.0)	34 (4.9)	78 (11.1)	
Grade 9	291 (41.5)	65 (9.3)	132 (18.8)	94 (13.4)	
Grade 10	256 (36.5)	53 (7.6)	138 (19.7)	65 (9.3)	
Hometown of Students					<0.001
Village	211 (30.1)	77 (11.0)	37 (5.3)	97 (13.8)	
Town	490 (69.9)	83 (11.8)	267 (38.1)	140 (20.0)	
Father’s occupation					<0.001
Farmers	102 (14.6)	31 (4.4)	11 (1.6)	60 (8.6)	
Businessman	76 (10.8)	29 (4.1)	39 (5.6)	8 (1.1)	
Government employee	127 (18.1)	30 (4.3)	75 (10.7)	22 (3.1)	
Private/corporate employee	112 (16.0)	17 (2.4)	86 (12.3)	9 (1.3)	
Military	209 (29.8)	27 (3.9)	69 (9.8)	113 (16.1)	
Others	75 (10.7)	26 (3.7)	24 (3.4)	25 (3.6)	
Mother’s occupation					<0.001
Farmers	103 (14.7)	32 (4.7)	11 (1.6)	60 (8.6)	
Businesswoman	64 (9.1)	25 (3.6)	33 (4.7)	6 (0.9)	
Government employee	59 (8.4)	12 (1.7)	40 (5.7)	7 (1.0)	
Private/corporate employee	27 (3.9)	3 (0.4)	22 (3.1)	2 (0.3)	
Military	2 (0.3)	0 (0.0)	1 (0.1)	1 (0.1)	
Housewife	431 (61.5)	83 (11.8)	190 (27.1)	158 (22.5)	
Others	15 (2.1)	5 (0.7)	7 (1.0)	3 (0.4)	
Dog ownership					<0.001
No	484 (69.0)	89 (12.7)	221 (31.5)	174 (24.8)	
Yes	217 (31.0)	71 (10.1)	83 (11.8)	63 (9.0)	

**Table 2:** Characteristics and management of dogs owned by students’ households in three rabies endemic towns of Bhutan (*n* = 217; Phuntsholing, Gelephu, Garpowoong).

Characteristics of Dog Owning Students		School			χ^2^ *p*-Value
	Total	GMSS	PMSS	GaMSS	
Dog source					*p* < 0.001
Adopted from street	31 (14.3)	8 (3.7)	8 (3.7)	15 (6.9)	
Given by neighbor/friends	115 (53.0)	28 (12.9)	54 (24.9)	33 (15.2)	
Purchased within Bhutan	28 (12.9)	12 (5.5)	11 (5.1)	5 (2.3)	
Purchased from outside country	4 (1.8)	0 (0.0)	4 (1.8)	0 (0.0)	
I don’t know	37 (17.1)	23 (10.6)	5 (2.3)	9 (4.2)	
Missing	2 (0.9)	0 (0.0)	1 (0.5)	1 (0.5)	
Dog number					*p* = 0.048
One dog	154 (71.0)	43 (19.8)	65 (30.0)	46 (21.2)	
More than one dog	63 (29.0)	28 (1.9)	18 (8.3)	17 (7.8)	
Dog keeping practices					*p* < 0.001
Free roaming all the time	54 (24.9)	24 (11.1)	14 (6.5)	16 (7.4)	
Keep inside house compound all the time	94 (43.3)	26 (12.0)	52 (24.0)	16 (7.4)	
Roam freely outside during day-time	65 (30.0)	21 (9.7)	16 (7.4)	28 (12.9)	
Roam freely during night-time	2 (0.9)	0 (0.0)	0 (0.0)	2 (0.9)	
Missing	2 (0.9)	0 (0.0)	1 (0.5)	1 (0.5)	
Dog vaccination status *					*p* = 0.009
No	26 (12.0)	14 (6.5)	4 (1.8)	8 (3.7)	
Yes	163 (75.1)	45 (20.7)	67 (30.9)	51 (23.5)	
I don’t know	26 (12.0)	12 (5.5)	11 (5.1)	3 (1.4)	
Missing	2 (0.9)	0 (0.0)	1 (0.5)	1 (0.5)	
Dog sterilized *					*p* = 0.239
No	89 (41.0)	33 (15.2)	26 (12.0)	30 (13.8)	
Yes	73 (33.6)	24 (11.1)	30 (13.8)	19 (8.8)	
I don’t know	53 (24.4)	14 (6.5)	26 (12.0)	13 (6.0)	
Missing	2 (0.9)	0 (0.0)	1 (0.5)	1 (0.5)	

Note: * The frequency and percentage are based on the student’s responses.

**Table 3:** Characteristics of dog bites and health-seeking behaviors among students bitten by dogs (*n* = 111).

Variables		Schools			χ^2^ *p*-Value
	Total	GMSS	PMSS	GaMSS	
What type of dog bit you?					*p* = 0.59
Pet dog	58 (52.3)	15 (13.5)	31 (27.9)	12 (10.8)	
Stray dog	44 (39.6)	7 (6.3)	27 (24.3)	10 (9.0)	
I don’t know	5 (4.5)	0 (0.0)	4 (3.6)	1 (0.9)	
Missing	4 (3.6)	0 (0.0)	2 (1.8)	2 (1.8)	
What was the reason for the bite?					*P* = 0.006
Provoked bite	43 (38.7)	13 (11.7)	27 (24.3)	3 (2.7)	
Unprovoked bite	64 (57.7)	9 (8.1)	35 (31.5)	20 (18.0)	
Missing	4 (3.6)	0 (0.0)	2 (1.8)	2 (1.8)	
What happened to the biting dog within three month after the bite?					*p* = 0.091
Died	18 (16.2)	6 (5.4)	9 (8.1)	3 (2.7)	
Disappeared	9 (8.1)	0 (0.0)	7 (6.3)	2 (1.8)	
dog still alive	56 (50.5)	12 (10.8)	28 (25.2)	16 (14.4)	
The dog was killed	1 (0.9)	1 (0.9)	0 (0.0)	0 (0.0)	
I don’t know	23 (20.7)	3 (2.7)	18 (16.2)	2 (1.8)	
Missing	4 (3.6)	0 (0.0)	2 (1.8)	2 (1.8)	
What did you do to the bite wound?					*p* = 0.130
Applied antiseptics to the wound	5 (4.5)	1 (0.9)	4 (3.6)	0 (0.0)	
Applied local herbs/medicine	13 (11.7)	7 (6.3)	3 (2.7)	3 (2.7)	
Washed bite wound with soap and water	69 (62.2)	11 (9.9)	41 (36.9)	17 (15.3)	
Washed bite wound with water only	12 (10.8)	2 (1.8)	8 (7.2)	2 (1.8)	
I did nothing	8 (7.2)	1 (0.9)	6 (5.4)	1 (0.9)	
Missing	4 (3.6)	0 (0.0)	2 (1.8)	2 (1.8)	
Have you visited a hospital after the bite?					*p* = 0.098
Yes	94 (84.7)	22 (19.8)	51 (46.0)	21 (18.9)	
No	13 (11.7)	0 (0.0)	11 (9.9)	2 (1.8)	
Missing	4 (3.6)	0 (0.0)	2 (1.8)	2 (1.8)	
Did you receive rabies vaccine injections?					*p* = 0.230
Yes	92 (82.9)	21 (18.9)	50 (45.1)	21 (18.9)	
No	15 (13.5)	1 (0.9)	12 (10.8)	2 (1.8)	
Missing	4 (3.6)	0 (0.0)	2 (1.8)	2 (1.8)	

**Table 4:** Student level of knowledge regarding rabies and perception towards dog bite management and rabid dog.

Knowledge on Rabies					
Variable	Frequency (%)	School Name			χ^2^
		GMSS	PMSS	GaMSS	*p*-Value
Knowledge on causes of rabies					
Psychological	189 (27.5)	47 (6.8)	78 (11.3)	64 (9.3)	0.678
Associated with spirit	28 (4.1)	6 (0.9)	7 (1.0)	15 (2.2)	0.065
Virus	399 (58.0)	99 (14.4)	150 (21.8)	150 (21.8)	0.002
Starvation and thirst	109 (15.8)	31 (4.5)	47 (6.8)	31 (5.5)	0.208
Bacteria	326 (47.4)	78 (11.3)	138 (20.1)	110 (16.0)	0.756
Eating bad food or poison	256 (37.2)	62 (9.0)	110 (16.0)	84 (12.2)	0.723
I don’t know	87 (12.7)	16 (2.3)	55 (8.0)	16 (2.3)	<0.001
Knowledge on susceptible host of rabies					
Bat	140 (20.4)	47 (6.8)	49 (7.1)	44 (6.4)	0.002
Wild birds	27 (3.9)	7 (1.0)	15 (2.2)	5 (0.7)	0.208
Dog	651 (94.6)	147 (21.4)	280 (40.7)	224 (32.7)	0.843
Domestic animals (cow, pig, horse etc.)	275 (40.0)	52 (7.6)	80 (11.6)	43 (12.4)	<0.001
Cat	258 (37.5)	70 (10.2)	103 (15.0)	85 (12.4)	0.091
Insects	9 (1.3)	1 (0.1)	6 (0.9)	2 (0.3)	0.509
Poultry	16 (2.3)	3 (0.4)	12 (1.7)	1 (0.2)	0.014
Snake	19 (2.8)	6 (0.9)	9 (1.3)	4 (0.6)	0.396
Wild cats (tiger, leopard etc.)	97 (14.1)	34 (4.9)	38 (5.5)	25 (3.6)	0.006
Knowledge on mode of transmission of rabies					
Consumption of cooked meats	68 (9.9)	10 (1.5)	20 (2.9)	38 (5.5)	<0.001
Consumptions of milk products	101 (14.7)	14 (2.0)	17 (2.5)	70 (10.2)	<0.001
Contact with dog urine and feces	190 (27.6)	33 (4.8)	88 (12.8)	69 (10.0)	0.121
Contact with saliva over broken skin	176 (25.6)	34 (4.9)	62 (9.0)	80 (11.6)	0.001
Dog bites	603 (87.7)	144 (20.9)	244 (35.5)	215 (31.3)	<0.001
From contaminated water	23 (3.3)	7 (1.0)	6 (0.9)	10 (1.5)	0.241
From contaminated soil	8 (1.2)	3 (0.4)	4 (0.6)	1 (0.2)	0.383
Scratches of animals	297 (43.2)	77 (11.2)	127 (18.5)	93 (13.5)	0.158
Knowledge on clinical signs of rabies in dog					
Aggressiveness and tendency to bite	470 (68.3)	111 (16.1)	215 (31.3)	144 (20.9)	0.016
Coughing	102 (14.8)	22 (3.2)	46 (6.7)	34 (4.9)	0.907
Diarrhea	60 (8.7)	14 (2.0)	31 (4.5)	15 (2.2)	0.256
Excessive salivation	262 (38.1)	74 (10.8)	94 (13.7)	94 (13.7)	0.003
Fear of water	341 (49.6)	87 (12.6)	112 (16.3)	142 (20.6)	<0.001
Paralysis of leg	103 (15.0)	23 (3.3)	49 (7.1)	31 (4.5)	0.567
Resting in the shade	98 (14.2)	21 (3.1)	49 (7.1)	28 (4.1)	0.308
Perception on post bite cares and rabid dogs					
What should you do if you are bitten by dogs?					
Wash with soap and water for 15 min	598 (86.9)	124 (18.0)	259 (37.6)	215 (31.3)	0.003
Go to the hospital and get vaccination	635 (92.3)	149 (21.7)	266 (38.7)	220 (32.0)	0.05
Do the local treatment	65 (9.5)	14 (2.0)	25 (3.6)	26 (3.8)	0.569
Do nothing and allow wound to heal	5 (0.7)	2 (0.3)	2 (0.3)	1 (0.1)	0.726
What will you do if you see dog with aggressive behavior?					
Kill the dog	23 (3.3)	8 (1.2)	7 (1.0)	8 (1.2)	0.008
Report to teachers	104 (15.1)	34 (4.9)	48 (3.2)	22 (3.2)	0.002
Report to livestock officers	427 (62.1)	108 (15.7)	169 (21.8)	150 (21.8)	0.029
Take for treatments to animal hospital	472 (68.6)	108 (15.7)	197 (28.6)	167 (24.3)	0.496
Do nothing	72 (10.5)	14 (2.0)	43 (6.3)	15 (2.2)	0.008

**Table 5:** Logistic regression model showing the variables associated with the knowledge and perception level of the students on rabies (*n* = 688).

Knowledge Level of Students on Rabies								
Variables	Category	Adequate Knowledge		Total	Univariable Analysis	*p*-Value	Multivariable Analysis	*p*-Value
		No	Yes		OR (95%CI)		Adjusted OR (95%CI)	
School	GMSS	78	78	156	Reference	Reference	Reference	Reference
	PMSS	192	105	297	0.6 (0.4–0.8)	0.003	0.7 (0.4–1.1)	0.001
	GaMSS	141	94	235	0.7 (0.4–1.0)	0.051	0.6 (0.3–0.8)	0.039
Grade in which student study	Grade 8	97	54	151	Reference	Reference	Reference	Reference
	Grade 9	177	107	284	1.1 (0.7–1.6)	0.694	1.1 (0.8–1.8)	0.535
	Grade 10	137	116	253	1.5 (1.0–2.3)	0.047	1.7 (1.1–2.6)	0.02
Attended rabies awareness program	No	268	151	419	Reference	Reference	Reference	Reference
	Yes	143	126	269	1.6 (1.2–2.1)	0.017	1.5 (1.1–2.1)	0.009
Sex	Female	236	164	400	Reference	Reference	Reference	Reference
	Male	175	113	288	0.9 (0.7–1.3)	0.641	0.9 (0.7–1.3)	0.857
Age	Adolescent	132	96	288	Reference	Reference		
	Young	279	181	460	0.9 (0.6–1.2)	0.018		
**Perception Level of Students on Rabies**								
**Variables**	**Category**	**Favorable Perception**		**Total**	**Univariable Analysis**		**Multivariable Analysis**	***p*-Value (Multivariable Analysis)**
		**No**	**Yes**		**OR (95%CI)**		**Adjusted OR (95%CI)**	
Mother occupation	Self employed	264	339	603	Reference	Reference	Reference	Reference
	Employed	27	58	85	1.7 (1.0–2.8)	0.037	1.7 (1.0–2.8)	0.031
Grade in which student study	Grade 8	72	79	151	Reference	Reference	Reference	Reference
	Grade 9	138	146	284	1.0 (0.7–1.4)	0.857	1.00 (0.7–1.5)	0.881
	Grade 10	81	172	253	1.9 (1.3–2.9)	0.002	1.9 (1.3–3.2)	0.001
Sex	Female	169	231	400	Reference	Reference	Reference	Reference
	Male	122	166	288	1.0 (0.7–1.4)	0.977	1.0 (0.7–1.3)	0.848
School	GMSS	63	93	156	Reference	Reference	Reference	Reference
	PMSS	129	168	297	0.9 (.06–1.3)	0.533	0.8 (0.5–1.3)	0.401
	GaMSS	99	136	235	0.9 (0.6–1.4)	0.976	1.0 (0.7–1.5)	0.937
Age	Adolescent	132	96	288	Reference	Reference		
	Young	279	181	460	0.7 (0.5–1.0)	<0.001		
Hometown of the students	Town	213	267	480	Reference	Reference		
	Village	78	130	208	1.3 (1.0–1.9)	0.094		

## Data Availability

Supplementary Materials, Table S2: Knowledge, Attitude and Practice survey data.

## Supplementary Materials

The following are available online at https://www.mdpi.com/2414-6366/6/1/28/s1, Table S1: Scoring system, Table S2: Knowledge, Attitude and Practice survey data.
